# HEALTHCARE DISPARITY IN THE ASSESSMENT OF DEMENTIA IN THE PRIMARY CARE SETTING

**DOI:** 10.1093/geroni/igac059.1901

**Published:** 2022-12-20

**Authors:** Regina Gibson, Maneetha Kodali, Priya Priyambada, Priya Mendiratta, Danish Hasan, Melodee Harris, Jeanne Wei, Gohar Azhar

**Affiliations:** University of Arkansas for Medical Sciences, Little Rock, Arkansas, United States; University of Arkansas for Medical Sciences, Little Rock, Arkansas, United States; University of Arkansas for Medical Sciences, Little Rock, Arkansas, United States; University of Arkansas for Medical Sciences, Little Rock, Arkansas, United States; University of Arkansas for Medical Sciences, Little Rock, Arkansas, United States; University of Arkansas for Medical Sciences, Little Rock, Arkansas, United States; University of Arkansas for Medical Sciences, Little Rock, Arkansas, United States; University of Arkansas for Medical Sciences, Little Rock, Arkansas, United States

## Abstract

Literature suggests racial implicit bias can significantly affect quality of care. Data demonstrates that older adult Blacks are twice as likely to have dementia as their White counterpart. Inappropriate cognitive screening by providers or diagnosing dementia just based on clinical impression could easily result in an over or under-estimation of the dementia. We reviewed the rate and method of cognitive screening in randomly selected patient records (n=75) at a primary care university clinic. Our results indicated that the cognitive screening rate for Black patients was lower compared to their White counterpart (43.4% Blacks vs 68.5% Whites, p < 0.05). We designed a quality improvement project to identify any contributory causes and challenges involved in screening for dementia with the goal to reduce racial disparity in dementia diagnosis. We identified knowledge deficits in providers in their approach towards patients with dementia and a lack of experience in their use of appropriate instruments for cognitive screening. A multipronged educational program, with videos, case conferences, presentations and one-to-one training by dementia experts and a neuropsychologist was employed to reduce the bias and train the providers in appropriate screening methods. Post-educational intervention, screening rates greatly improved in n=75 randomly selected patients from both races. In Whites, the screening rate increased by 20.9% to 89.4% and in Blacks by 38.9% to 82.3% (p < 0.05). Overall, the quality improvement driven educational intervention improved the self-efficacy of providers and improved the standardized dementia screening rates in Black patients to levels comparable to those of White patients.

